# Testosterone Deficiency Accelerates Neuronal and Vascular Aging of SAMP8 Mice: Protective Role of eNOS and SIRT1

**DOI:** 10.1371/journal.pone.0029598

**Published:** 2012-01-04

**Authors:** Hidetaka Ota, Masahiro Akishita, Takuyu Akiyoshi, Tomoaki Kahyo, Mitsutoshi Setou, Sumito Ogawa, Katsuya Iijima, Masato Eto, Yasuyoshi Ouchi

**Affiliations:** 1 Department of Geriatric Medicine, Graduate School of Medicine, University of Tokyo, Bunkyo-ku, Tokyo, Japan; 2 Hamamatsu University School of Medicine, Department of Molecular Anatomy, Hamamatsu, Shizuoka, Japan; University of Padova, Medical School, Italy

## Abstract

Oxidative stress and atherosclerosis-related vascular disorders are risk factors for cognitive decline with aging. In a small clinical study in men, testosterone improved cognitive function; however, it is unknown how testosterone ameliorates the pathogenesis of cognitive decline with aging. Here, we investigated whether the cognitive decline in senescence-accelerated mouse prone 8 (SAMP8), which exhibits cognitive impairment and hypogonadism, could be reversed by testosterone, and the mechanism by which testosterone inhibits cognitive decline. We found that treatment with testosterone ameliorated cognitive function and inhibited senescence of hippocampal vascular endothelial cells of SAMP8. Notably, SAMP8 showed enhancement of oxidative stress in the hippocampus. We observed that an NAD^+^-dependent deacetylase, SIRT1, played an important role in the protective effect of testosterone against oxidative stress-induced endothelial senescence. Testosterone increased eNOS activity and subsequently induced SIRT1 expression. SIRT1 inhibited endothelial senescence via up-regulation of eNOS. Finally, we showed, using co-culture system, that senescent endothelial cells promoted neuronal senescence through humoral factors. Our results suggest a critical role of testosterone and SIRT1 in the prevention of vascular and neuronal aging.

## Introduction

Advancing age is the most significant risk factor for the development of cognitive impairment [1], [2]; however, what age-related changes underlie this effect remains uncertain. With advancing age, men experience a significant decrease in the circulating level of testosterone. Although studies have shown alterations in mood, libido, and cognition resulting from testosterone deficiency [3], the full range of consequences of age-related testosterone loss remains incompletely defined. In a small clinical study of men recently diagnosed with cognitive impairment, testosterone treatment improved performance on cognitive tests [4]. In a prospective longitudinal study using subjects from the Baltimore Longitudinal Study on Aging, men who developed Alzheimer disease (AD) were observed to exhibit low testosterone levels 5–10 years prior to the clinical diagnosis of AD [5]. With a relationship between age-related testosterone decline in men and increased risk for cognitive impairment reasonably well established, a critical issue is how testosterone contributes to the pathogenesis of cognitive decline with aging. The most likely hypothesis is through the regulation of accumulation of amyloid ß (Aß) peptides, which are widely believed to be the critical initiating step in the pathogenesis of AD. However, it is becoming increasingly clear that not all aspects of cognitive decline can be explained by Aß [6], [7]. Findings from such diverse lines of investigations as neuroimaging and clinical trials suggest that non-Aß factors also contribute to memory deficit in aged men.

In *S. cerevisiae*, the *Sir2* (silent information regulator-2) family of genes governs budding exhaustion and replicative life span [8], [9]. *Sir2* has been identified as an NAD^+^-dependent histone deacetylase and is responsible for maintenance of chromatin silencing and genome stability. Mammalian sirtuin 1 (*Sirt1*), the closest homolog of *Sir2*, regulates the cell cycle, senescence, apoptosis and metabolism, by interacting with a number of molecules such as p53. As recently reported, overexpression of SIRT1 in the brain improved the memory deficit in a mouse model of AD via activation of the transcription of α-secretase [10].

An increasing body of evidence suggests the presence of a link between cognitive decline and vascular dysfunction, especially atherosclerosis [11]. Senescence of endothelial cells is involved in endothelial dysfunction and atherogenesis, and SIRT1 has been recognized as a key regulator of vascular endothelial homeostasis, controlling angiogenesis, endothelial senescence, and dysfunction [12]–[14].

In the present study, we demonstrated that cognitive impairment in senescence-accelerated mouse prone 8 (SAMP8), a model of cognitive decline with aging, is associated with endothelial senescence in the hippocampus and is ameliorated by testosterone replacement. SIRT1 plays an important role in prevention of endothelial senescence induced by oxidative stress [13]. We suggest that the protection against endothelial senescence in the hippocampus through up-regulation of testosterone and SIRT1 could contribute to a novel therapeutic strategy against cognitive decline with aging.

## Results

### Treatment with dihydrotestosterone ameliorated cognitive function of SAMP8

**Figure 1:**
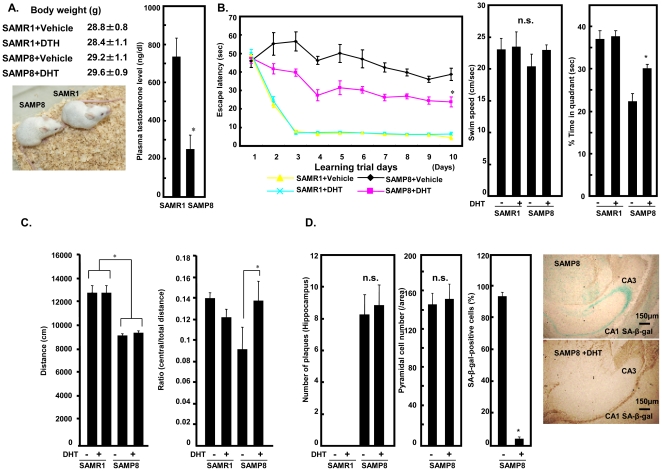
Testosterone deficiency causes senescence of hippocampus and cognitive impairment in SAMP8 mice. **A.** Body weight, appearance, and plasma testosterone level of male SAMR1 and SAMP8 mice at 12 weeks of age. **B.** Escape latency of SAMR1 (N = 10) and SAMP8 mice (N = 10). Male mice were treated daily for 2 weeks with DHT (500 µg s.c) before trials. Swim speed during quadrant test on day 10. **C.** Total distance and the ratio of central/total distance were measured in open field tests. **D.** Number of amyloid ß plaques, pyramidal cells, and SA-ßgal-positive cells in CA1 and CA3 areas of hippocampus in SAMR1 and SAMP8. (*p<0.05, n.s: not significant).

In order to assess the effects of testosterone on cognitive function, we used an in vivo model of aging, SAMP8, and a control counterpart strain, SAMR1. SAMP8 was originally derived from AKR/J strain, litters of which show the characteristic of cognitive decline with aging. These mice exhibit age-related deficits in learning and memory at an early age, and are considered a suitable animal model to study aging and memory deficit. Body weight, appearance, and plasma testosterone level of SAMR1 and SAMP8 at 12 weeks of age were determined. Body weight and appearance did not differ between SAMR1 and SAMP8, but plasma testosterone level in SAMP8 was lower than that in SAMR1 (Figure 1A). By determining the time required to find the platform (escape latency) as a function of days of training in the Morris water maze, we observed a marked decline in performance in SAMP8 compared with SAMR1 (Figure 1B). Because testosterone acts in part through aromatase-dependent conversion to estradiol, non-aromatizable dihydrotestosterone (DHT) was used to examine a direct role of androgens through androgen receptor (AR). SAMP8 treated with DHT showed significantly reduced escape latency time compared with untreated SAMP8. There was no difference in swim speed between the groups; however, % time in the quadrant was increased in DHT-treated SAMP8 (Figure 1B). These results indicate that DHT treatment ameliorated cognitive dysfunction in SAMP8. The water-maze is appropriate for hippocampal-dependent paradigms. However, DHT administration may affect behavior and how animals respond to different stimuli. Therefore, we performed an open field test to examine locomotion, exploratory behavior, and anxiety. No significant effect of DHT on locomotor performance was observed in SAMR1 and SAMP8, whereas SAMR1 moved significantly more compared with SAMP8 (Figure 1C). The ratio of the distance travelled in the central area to that in the total area in the open- field, an indirect measure of exploratory behavior and anxiety [15], was also observed. In SAMP8, DHT increased this ratio (Figure 1C), suggesting that DHT promoted exploratory behavior and diminished anxiety.

Next, we assessed the number of amyloid ß plaques, pyramidal cells, and SA-ßgal-positive cells in CA1 and CA3 areas of the hippocampus in these mice (Figure 1D). The number of plaques was increased in SAMP8 compared with SAMR1, but was unaltered by treatment with DHT. The number of SA-βgal-stained cells was significantly increased in SAMP8 compared with SAMR1, but treatment with DHT prevented this in SAMP8 despite no difference in pyramidal cell number (Figure 1D).

### DHT treatment increased protein and mRNA expression of SIRT1 in SAMP8

**Figure 2:**
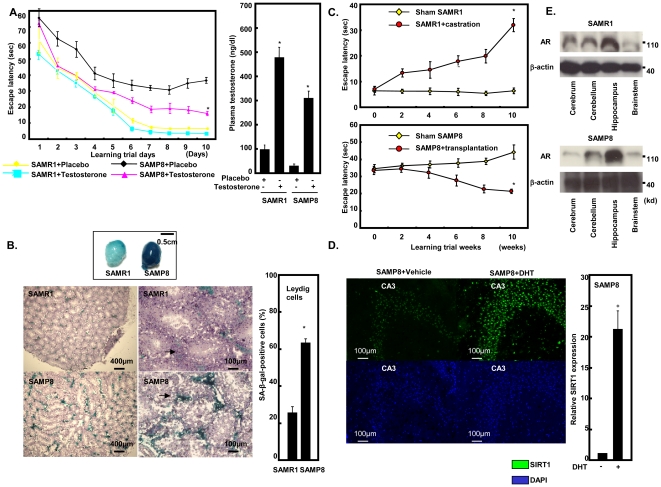
Supplementation of testosterone improves cognitive function in SAMP8 mice. **A.** Escape latency and plasma testosterone level of male SAMR1 (N = 10) and SAMP8 mice (N = 10) at 18 months of age. These mice were implanted subcutaneously with a placebo or a 21-day-release 2.5 mg testosterone pellet in the dorsal neck. **B.** Number of SA-βgal-stained Leydig cells in testes in SAMR1 and SAMP8. Arrows indicate Leydig cells. Representative SA-βgal-stained testes from SAMR1 and SAMP8. **C.** Escape latency of castrated SAMR1 (upper, N = 5) and recipient SAMP8 (lower, N = 5). Observation (0–10 weeks) was started from 3 weeks after operation. **D.** SIRT1 expression in hippocampus of SAMP8 with or without DHT treatment. Immunofluorescent staining for SIRT1 (green) and DAPI (blue). **E.** Expression of AR in SAMR1 and SAMP8 brains. (*p<0.05).

Furthermore, to estimate the role of testosterone deficiency in SAMP8, we examined the effect of testosterone supplementation on cognitive function in much older SAMR1 and SAMP8. Similarly to young mice, we observed a marked decline in performance in SAMP8 compared with SAMR1 at 18 months of age. SAMP8 implanted with testosterone pellets showed significantly reduced escape latency time compared with placebo-treated SAMP8 (Figure 2A). Plasma testosterone level in SAMP8 at 18 months of age was lower than that in SAMR1, but implanted mice showed recovery to the level in young mice (Figure 2A). These results indicated that similar to DHT, testosterone also showed the improvement of cognitive function in SAMP8. Next, we examined the cause of low plasma testosterone in SAMP8. SAMP8 showed no testicular atrophy (Figure S1A), but more senescent phenotypes in Leydig cells, which produce testosterone in testes, than SAMR1 (Figure 2B). Moreover, we tried to allotransplant testes from SAMR1 to SAMP8 (Figure S1B). Although performance gradually responded to treatment up to 8–10 weeks, castrated SAMR1 showed a marked decline in performance whereas recipient SAMP8 showed cognitive improvement (Figure 2C).

As recently reported, overexpression or activation of SIRT1 inhibits cellular senescence and protects cellular function in various cell lines [13], [16]. Therefore, we examined SIRT1 expression in the hippocampus of SAMP8 with or without DHT treatment, at 12 weeks of age. DHT treatment increased the protein and mRNA expression of SIRT1 in SAMP8 (Figure 2D). To investigate further the involvement of AR, we examined the expression of AR in SAMR1 and SAMP8 brains. The expression of AR was more abundant in the hippocampus than in other brain regions of SAMR1 and SAMP8 (Figure 2E).

### Oxidative stress was increased in hippocampal cells of SAMP8

**Figure 3:**
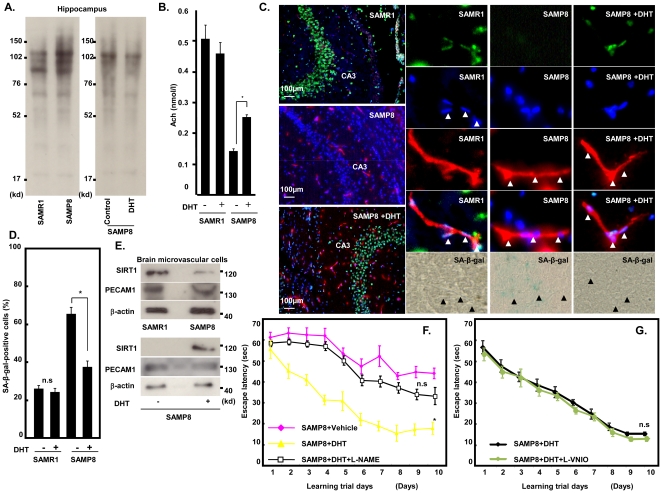
Senescent endothelial cells of hippocampus are decreased by treatment with DHT. **A.** Oxidative stress level was measured by detection of carbonyl groups introduced into proteins. **B.** Acetyl-choline concentration was measured by a colorimetric method. **C.** SA-βgal-stained endothelial cells and SIRT1 expression in CA3 area of hippocampus in SAMR1 and SAMP8 with or without DHT treatment. Immunofluorescent staining for SIRT1 (green), PECAM-1 (red), and DAPI (blue). **D.** Number of SA-βgal-stained endothelial cells in CA3 area of hippocampus in SAMR1 and SAMP8 with or without DHT treatment. **E.** Expression of SIRT1, PECAM-1, and β-actin was analyzed using cerebral micro vascular cells. **F.** Escape latency of SAMR1 (N = 10) and SAMP8 mice (N = 10). Male mice were treated daily for 2 weeks with DHT (500 µg s.c) and L-NAME (20 mg/kg gavage) before trials. **G.** Escape latency of SAMR1 (N = 5) and SAMP8 mice (N = 5). Male mice were treated daily for 2 weeks with DHT (500 µg s.c) and L-VNIO (5 mg/kg IP) before trials. (*p<0.05, n.s: not significant).

Oxidative stress may be closely related to senescence and age-related diseases. Also, an increase in oxidative stress has been suggested to be one of the earliest pathological changes in the brain in conditions with cognitive impairment such as AD [17]. Then, we examined the level of oxidative stress, using the SAMR1 and SAMP8 hippocampus at 12 weeks of age. SAMP8 hippocampus showed an increase in the level of oxidative stress compared with SAMR1 as judged by detection of carbonylated proteins. DHT treatment decreased carbonylated proteins in the SAMP8 hippocampus (Figure 3A). In parallel, the concentration of the neurotransmitter acetylcholine in hippocampal lysates was decreased in SAMP8 compared with that in SAMR1, and DHT treatment prevented this (Figure 3B).

Testosterone and DHT acts on vascular endothelial cells and stimulates the PI3K/Akt pathway, leading to eNOS activation through direct interaction of AR [18], [19]. The eNOS/SIRT1 axis is recognized as one of the fundamental determinants of endothelial senescence, and SIRT1 acts as a driver of cellular stress resistance [20]. To examine the influence of DHT treatment on endothelial cells, we determined the degree of senescence and the expression of SIRT1 in endothelial cells around the CA3 area of the hippocampus. DHT-treated SAMP8 showed a reduction of SA-βgal-stained endothelial cells and increased SIRT1 expression compared to untreated SAMP8 (Figure 3C and D). To confirm that these cells were endothelial cells, not neuronal cells, cerebral microvessels were isolated from SAMR1 and SAMP8. In parallel with immunohistological staining, SAMP8 showed a reduction of SIRT1 expression compared to SAMR1, and DTH treatment increased SIRT1 expression compared to that in untreated SAMP8 (Figure 3E). These results suggest that vascular endothelial senescence in the hippocampus may be related to the memory deficit in SAMP8. Since testosterone and DHT activates eNOS, a NOS inhibitor, *N^G^*-nitro-L-arginine methyl ester hydrochloride (L-NAME), and N^5^-(1-lmino-3-butenyl)-L-ornithine (L-VNIO), a selective neuronal NOS (nNOS) inhibitor, were applied to examine the involvement of NOS in this process. L-NAME abrogated the effects of DHT on cognitive function (Figure 3F). In contrast, L-VNIO did not change the effect of DHT (Figure 3G). These results suggest that eNOS/SIRT1 in endothelial cells may play an important role in the protective effect of testosterone against senescence of the hippocampus.

### SIRT1 plays an important role in the protective effect of testosterone against endothelial senescence

**Figure 4:**
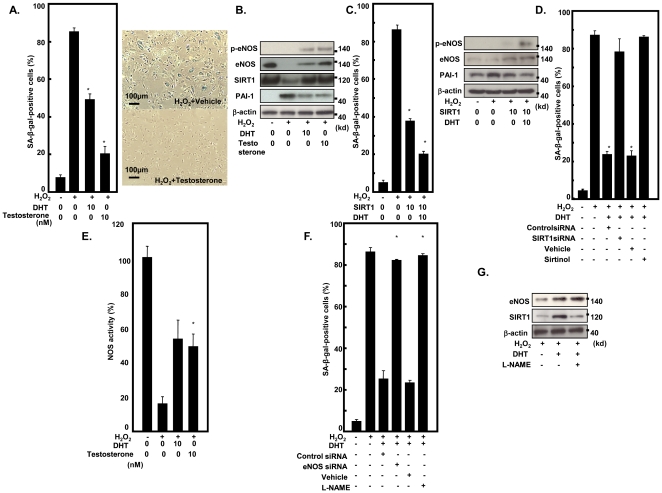
Testosterone inhibits oxidative stress-induced endothelial senescence through eNOS/SIRT1. **A.** Testosterone inhibited SA-βgal activity and senescent morphological appearance induced by hydrogen peroxide (100 µmol/L). **B.** Expression of eNOS, SIRT1, and PAI-1 in hydrogen peroxide (100 µmol/L)-treated HUVEC under treatment with DHT or testosterone. **C.** Overexpression of SIRT1 and DHT reduced SA-βgal activity. eNOS expression was increased by overexpression of SIRT1, and DHT increased phosphorylation of eNOS (Ser1177). **D.** SIRT1 inhibition by siRNA or sirtinol (100 µmol/L) abrogated the effect of testosterone on SA-βgal activity. **E.** Treatment with testosterone or DHT increased eNOS activity. **F.** eNOS inhibition by siRNA or L-NAME (10 mM) abrogated the effect of testosterone on SA-βgal activity. **G.** Treatment with L-NAME decreased SIRT1 expression in DHT-treated HUVEC. (*p<0.05, N = 3).

Following the animal experiments, we examined whether testosterone inhibited endothelial senescence *in vitro* using cultured cells. We induced premature endothelial senescence by addition of H_2_O_2_ 100 µmol/L for 1 hour. DHT or testosterone treatment inhibited SA-βgal activity and the morphological appearance of senescence (Figure 4A). We observed that oxidative stress decreased eNOS and SIRT1 and increased PAI-1 expression, and DHT or testosterone treatment prevented these changes and increased the phosphorylation of eNOS at Ser1177 (Figure 4B). Overexpression of SIRT1 significantly inhibited oxidative stress-induced senescence, and DHT accelerated the effect of SIRT1 through phosphorylation of eNOS at Ser1177 (Figure 4C). To determine the role of endogenous SIRT1, DHT-treated endothelial cells were transfected with SIRT1 siRNA or treated with sirtinol, a chemical inhibitor of SIRT1. SIRT1 siRNA or sirtinol abrogated the effect of DHT on SA-βgal activity (Figure 4D). We previously reported that testosterone activated eNOS [18], and eNOS activation promoted SIRT1 expression [21]. Accordingly, we examined the role of eNOS in the protective effect of testosterone. We observed that DHT or testosterone treatment increased NOS activity that was reduced by oxidative stress (Figure 4E). Treatment with eNOS siRNA or L-NAME decreased the inhibitory effect of DHT on a senescent phenotype in parallel with SIRT1 expression (Figure 4F and G). These results indicate that eNOS/SIRT1 play an important role in the protective effect of testosterone and DHT against a senescent phenotype.

### Senescent endothelial cells induced by oxidative stress promoted neuronal senescence

**Figure 5:**
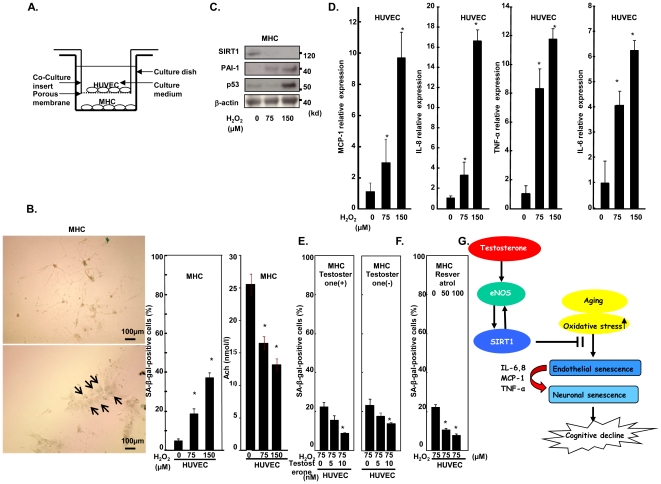
Oxidative stressed-induced endothelial cell senescence promotes adjacent neuronal cell senescence. **A.** Co-culture cell culture dish. **B.** Number of SA-βgal-stained MHC and senescent appearance of MHC were increased, and acetyl-choline concentration was decreased by co-culture with senescent endothelial cells. Senescent MHC are indicated by arrows. **C.** Expression of SIRT1, PAI-1, p53, and β-actin in MHC co-cultured with senescent endothelial cells. **D.** Expression of IL-6, IL-8, MCP-1, and TNF-α in endothelial cells were analyzed by RT-PCR. **E.** The number of SA-βgal-stained MHC was decreased by treatment with testosterone in both MHC and HUVEC (MHC, testosterone (+)), or HUVEC (MHC, testosterone (−)) alone. **F.** Resveratrol decreased the number of SA-βgal-stained MHC co-cultured with senescent endothelial cells. (*p<0.05, N = 3). **G.** Hypothetical signal transduction pathways of testosterone in endothelial cells.

Finally, we hypothesized that endothelial senescence promotes senescence of adjacent neuronal cells. To test this hypothesis, we used a co-culture system of endothelial cells (HUVEC) with neuronal cells (mouse hippocampal neuronal cells; MHC) (Figure 5A). Both cells were co-cultured, but were separated by a microporous polycarbonate membrane, for 10 days after endothelial cells were treated with hydrogen peroxide, and the senescent phenotype of MHC was analyzed. We found that the number of SA-βgal-positive cells and the senescent appearance of MHC were increased, and the concentration of acetylcholine in cells was decreased by co-culture with senescent endothelial cells (Figure 5B). In parallel with this, MHC showed increased PAI-1 and p53, and decreased SIRT1 expression (Figure 5C). We also found that senescent endothelial cells showed increased expression of inflammatory cytokines such as IL-6, IL-8, MCP-1, and TNF-α (Figure 5D). Both MHC and HUVEC, or HUVEC alone were treated with testosterone at 3 days before HUVEC were treated with hydrogen peroxide, and both cells were co-cultured for 10 days, and the senescent phenotype of MHC was analyzed. We found that the number of SA-βgal-positive MHC was decreased by treatment of HUVEC with testosterone irrespective of the treatment of MHC with testosterone (Figure 5E). In addition, we found that a SIRT1 activator, resveratrol treatment rescued the senescent phenotype of MHC (Figure 5F). These results suggest that senescent endothelial cells exhibit a senescence-associated secretory phenotype [22], induce neuronal senescence, and testosterone rescues it through up-regulation of SIRT1 (Figure 5G).

## Discussion

Testosterone level and cognitive function show a decline with age in men. A series of evidence suggests that this association is not just age related [23]. Results from cell culture and animal studies provide evidence that testosterone could have protective effects on brain function, especially in the hippocampus [24]. Here, we demonstrated that administration of testosterone restored cognitive function in male SAMP8 in association with improvement of the senescent phenotype in the hippocampus and cerebral vessels. We also showed that testosterone ameliorated endothelial senescence through eNOS/SIRT1-dependent mechanisms *in vitro*. The present study demonstrated that testosterone and SIRT1 interacts with each other and inhibited the senescence of hippocampal vascular and neuronal cells, suggesting that testosterone replacement therapy is a treatment option for cognitive decline with aging.

Testosterone may act in part through aromatase-dependent conversion to estradiol. To estimate a direct effect of androgens through AR, testosterone and DHT were used in this study. Both compounds showed significant protective effects on cognitive function.

In the present study, we used SAMP8 mice. SAMP is comprised of 14 strains derived from selective inbreeding of the AKR/J strain. SAMP8 exhibits age-related learning and memory deficits, as well as amyloid-like deposits in the brain [25]. Increased expression of hyperphosphorylated tau has also been detected in SAMP8 [26]. Given such features, SAMP8 has been proposed as a plausible age-associated AD animal model, and a suitable rodent model for studying the molecular mechanism underlying cognitive impairment [27]. A previous study has shown an age-related decrease in serum testosterone in SAMP8, and suggesting that impaired cognitive function in SAMP8 is due to reduced testosterone [28]. We observed that AR expression was abundant in the hippocampus of SAMR1 and SAMP8. Several studies have demonstrated that testosterone has a neuroprotective effect through AR in the hippocampus [29], [30], and testosterone induced NO productions via AR-dependent activation of eNOS in endothelial cells [18], [19].

Accumulating evidence suggests that NAD^+^-dependent deacetylase SIRT1 play an essential role for cellular senescence and cognitive function. SIRT1 modulates endothelial cellular senescence [13], and overexpression of SIRT1 exhibits neuroprotective effects in hippocampus, and cognitive function of *Sirt1*-KO mice is markedly impaired [10], [31], [32].

The precise etiologic mechanism of the cognitive decline with aging is unclear, but it has been identified that cardiovascular risk factors are associated with a higher incidence of cognitive impairment [33]. In addition, age-associated vascular inflammation is an early manifestation of chronic stress responses, i.e. overloading of ROS on endothelial cells [34]. Indeed, SAMP8 showed enhancement of oxidative stress and a senescent phenotype in the hippocampus. Notably, senescent endothelial cells were increased in the hippocampus of SAMP8 accompanied by a reduction of SIRT1, and L-NAME abrogated the effect of DHT on cognitive function. Therefore, we hypothesized that testosterone influenced cerebral endothelial senescence via eNOS/SIRT1, and that pro-inflammatory cytokines, which were derived from senescent endothelial cells, promoted senescence in adjacent neuronal cells. Indeed, we observed that testosterone induced eNOS activity, and subsequently increased SIRT1 expression in endothelial cells. Inhibition of eNOS/SIRT1 abrogated the effect of testosterone on endothelial senescence. In a co-culture system, we found that senescent endothelial cells promoted senescence of adjacent neuronal cells, and treatment of endothelial cells with testosterone inhibited senescence of adjacent neuronal cells. It can reasonably be speculated, therefore, that SIRT1 may exert salutary actions against cognitive decline with aging by preventing a senescence-associated secretory phenotype of endothelial cells. Because L-NAME is a non-selective inhibitor of NOS, it is possible that the effect of L-NAME might be in part a result of inhibition of nNOS in concert with eNOS. However, a specific nNOS inhibitor, L-VNIO did not change the effect of DHT in SAMP8. In co-culture experiments, we found that treatment with resveratrol or testosterone did not change the expression or activation of nNOS in MHC (Figure S1C and D). Further studies are needed to address the differential role of eNOS and nNOS, and the exact role of SIRT1 *in vivo*.

In conclusion, supplementation of testosterone prevented cognitive impairment of SAMP8, in which testosterone secretion was decreased in association with the senescence of testis Leydig cells, through an eNOS/SIRT1-dependent mechanism. Unprecedented reversal of the senescent hippocampal changes and vascular protection may justify exploration of a neuronal rejuvenation strategy by utilizing testosterone for the prevention of cognitive decline with aging, particularly through up-regulation of eNOS/SIRT1.

## Methods

### Materials

Dihydrotestosterone (DHT), testosterone, and *N^G^*-nitro-L-arginine methyl ester hydrochloride (L-NAME) were purchased from Sigma (St. Louis, MO). Hydrogen peroxide (H_2_O_2_) and resveratrol were purchased from Wako Pure Chemical Industries (Osaka, Japan). Testosterone and placebo pellets were purchased from Innovative Research of America (Sarasota, FL). N^5^-(1-lmino-3-butenyl)-L-ornithine (L-VNIO) was purchased from Enzo Life Sciences (Plymouth Meeting, PA).

### Cell culture

Human umbilical vein endothelial cells (HUVEC) were purchased from CAMBREX (Walkersville, MD). Population doubling levels (PDL) were calculated as described previously [35], and all experiments were performed at PDL of 10–11. In our preliminary experiments, HUVEC were cultured in EBM without phenol red (Clonetics, Walkersville,MD) with 10% dextran-charcoal-stripped serum to remove steroids from the culture medium. This condition, however, induced marked growth arrest and an increase in senescent cells. Consequently, we performed all experiments in EBM-2 (Clonetics) with 10% complete serum-supplemented medium.

### Animal experiments

The animal experiments were approved by our institutional review board (animal experiments ethics board, Graduate School of Medicine and Faculty of medicine, The university of Tokyo (approval ID: M-P-09-056)). Senescence-accelerated mice prone (SAMP) 8 and control senescence-accelerated mice resistant (SAMR) 1 male mice were all housed and maintained in a room at 22±2°C with automatic light cycles (12 h light/dark) and relative humidity of 40–60%. Mice were purchased from Japan SLC, Inc. (Shizuoka, Japan). Food and tap water were provided ad libitum throughout the study. In the water maze test of this study, a group of male SAMR1 (N = 10) and SAMP8 (N = 10) was first tested. Male mice of 12 weeks of age were treated daily for 2 weeks with DHT (500 µg in 0.05 ml/mouse) by subcutaneous injection (s.c.) in the neck before the water maze test. Male mice of 18 months of age underwent subcutaneously implantation of a placebo (N = 5) or a 21-day-release 2.5 mg testosterone (N = 5) pellet into the dorsal neck region. L-NAME was given by gavage once a day (20 mg/kg) [36]. L-VNIO was given by intraperitoneal injection (0.5 mg/kg) [37]. Small fragments of testis tissue fragments from SAMR1 were grafted under the back skin of castrated male SAMP8 as previously described [38]. Briefly, after removal of the capsule and obvious connective tissue, donor testes were cut into small fragments. Testis fragments were kept in Dulbecco's modified Eagle's medium (Gibco Lab Inc., Grand Island, NY, USA) on ice until grafting. SAMR1 were anesthetized and castrated, and testicular tissue fragments were grafted under the back skin of SAMP8. Mice were anesthetized with enflurane, killed by cervical dislocation, and trunk blood collected within 1 min. The blood was centrifuged and plasma testosterone was measured by radioimmunoassay method. The brain was removed for histological examination, after systemic perfusion with phosphate-buffered saline (PBS). For immunohistochemical studies, mouse brains were processed and labeled with anti-amyloid-β antibody (Immuno-Biological Laboratories Co., Ltd., Gunma, Japan) to visualize extracellular amyloid plaques, anti-NeuN antibody (Millipore, Billerica, MA) to assess pyramidal cell number, or DAPI (Dojindo Molecular Technologies, Inc., Tokyo, Japan) for nuclear staining. The primary antibody was purified rat anti-mouse CD31 (platelet endothelial cell adhesion molecule; PECAM-1) monoclonal antibody from Pharmingen (San Jose, CA, USA). Secondary antibodies (Alexa Fluor 488 donkey anti-rat IgG and Alexa Fluor 594 donkey anti-rat IgG) and antifade reagent were from Molecular Probes (Invitrogen). Fluorescent images were analyzed using a fluorescence microscope (BZ-9000, KEYENCE, Osaka, Japan).

### Plasmids and siRNA transfection

Proliferating cells were washed three times with growth medium and exposed to the indicated concentrations of testosterone or DHT diluted in medium. pIRES-SIRT1 plasmid was provided by Dr. M. Takata [39], and Dr. R.A. Weinberg [40]. Each plasmid was overexpressed by transfection using Lipofectamine LTX and PLUS reagents (Invitrogen) for HUVEC according to the manufacturer's instructions. Proliferating cells were transfected with each siRNA using silMPORTER (Upstate Cell Signaling Solutions). siRNAs for SIRT1 (GAT GAA GTT GAC CTC CTC A [41] and TGA AGT GCC TCA GAT ATT A), and eNOS were purchased from Santa Cruz Biotechnology, Inc.

### Immunoblotting and immunoprecipitation

Cells were lysed on ice for 1 hour in buffer (50 mmol/L Tris-HCl, pH 7.6, 150 mmol/L NaCl, 1% NP-40, 0.1% SDS, 1 mmol/L dithiothreitol, 1 mmol/L sodium vanadate, 1 mmol/L phenylmethylsulfonyl fluoride, 10 µg/mL aprotinin, 10 µg/mL leupeptin and 10 mmol/L sodium fluoride). Equal amounts of protein were separated by SDS/PAGE gel electrophoresis and transferred to nitrocellulose membranes. After blocking, the filters were incubated with the following antibodies; anti-SIRT1, anti-nNOS, anti-AR (Cell Signaling, Danvers, MA), anti-eNOS (BD Transduction Laboratories, San Jose, CA), anti-PAI-1 (Molecular Innovations, Southfield, MI), anti-PECAM-1 (Santa-Cruz Biotechnology, CA), and anti-ß-actin (Sigma). After washing and incubation with horseradish peroxidase-conjugated anti-rabbit or anti-mouse IgG (Amersham, Piscataway, NJ) for 1 hour, antigen-antibody complexes were visualized by using an enhanced chemiluminescence system (Amersham).

### Senescence-associated ß-galactosidase (SA-ßgal) staining

HUVEC were pretreated with diluted EGM-2 medium for 3 day. HUVEC were then washed three times with EGM-2 and treated for 1 hour with 100 µmol/l H_2_O_2_ diluted in EGM-2. After treatment, HUVEC were trypsinized, re-seeded at a density of 1×10^5^ in 60-mm dishes, and cultured with EGM-2 containing DHT or testosterone for 10 days. The proportion of SA-ßgal-positive cells was determined as described by Dimri et al [42].

### NOS activity assay

NOS activity was determined using an NOS assay kit (Calbiochem) according to the manufacturer's instructions.

### Measurement of acetylcholine

The concentration of acetylcholine was measured with a choline/acetylcholine quantification kit (BioVision, CA, USA) according to the manufacturer's instructions.

### Real-time quantitative reverse transcription PCR

Total RNA was isolated with ISOGEN (Nippon Gene Inc., Toyama, Japan). After treatment with Rnase-free Dnase for 30 min, total RNA (50 ng/µl) was reverse transcribed with random hexamers and oligo d(T) primers. The expression levels of SIRT1, IL-6, IL-8, MCP-1, and TNF-α relative to ß-actin were determined by means of staining with SYBR green dye and a LineGene fluorescent quantitative detection system (Bioflux Co., Tokyo, Japan). The following primers were used: SIRT1 F 5′-CCTGACTTCAGGTCAAGGGATGGTA-3′, R 5′-CTGATTAAAAATATCTCCTCGTACAG-3′; ß-actin F 5′-TGGGCATGGGTCAGAAGGAT-3′, R 5′-AAGCATTTGCGGTGGACCAT-3′; IL-6 F 5′-GGGAAGGTGAAGGTCGG-3′, R 5′-TGGACTCCACGACGTACTCAG-3′, IL-8 F 5′-CTGGCCGTGGCTCTCTTG-3′, R 5′-CCTTGGCAAAACTGCACCTTT-3′; TNF-α F 5′-GTAGCCCACGTCGTAGCAAAC-3′, R 5′-CTGGCACCACTAGTTGGTTGTC-3′; MCP-1 F 5′-CATTGTGGCCAAGGAGATCTG-3′, R 5′-CTTCGGAGTTTGGGTTTGCTT-3′.

### Co-culture system

For these experiments, co-culture dishes were used as outlined in Figure 5A. They were obtained from BD Biosciences (Erembodegem, Belgium) with a 6-well format. HUVEC were treated with H_2_O_2_ (100 µM) for 1 h and cultured on the permeable microporous (0.4 µm) membrane in the insert, and mouse hippocampus neuronal cells on the base of the culture dish, kept physically separated but allowing the passage of micromolecules through the porous membrane for 10 days. Mouse hippocampus neuronal cells were purchased from DS Pharma Biomedical Inc. (Osaka, Japan).

### Quantitative analysis of amyloid β

Measurement of amyloid β was performed using an amyloid β (1–40) (FL) assay kit (Immuno-Biological Laboratories Co., Ltd., Gunma, Japan) according to the manufacturer's instructions.

### Morris water maze test

The procedure of the Morris water maze test was described previously [43]. SAMR1 and SAMP8 mice were trained to find a visible platform with three trials on the first day, and then tested to find the hidden platform for 10 consecutive days. In each trial, the mice were allowed to swim until they found the hidden platform, or until 2 min had passed, and the mouse was then guided to the platform. On the test days, the platform was hidden 1 cm beneath the water. The escape latency was recorded by a video camera. The swim speed of each mouse was calculated by means of a video tracking system. Probe tests were performed on the 10^th^ day. During percent time quadrant test, the platform was removed from the pool. Mice were started in a position opposite the location of the platform position and allowed to swim for 60 seconds.

### Open field test

The open field test fear response to novel stimuli was used to assess locomotion, exploratory behavior, and anxiety. Open field test protocols were modified from that of Lukacs et al [44]. The open field test consisted of a wooden box (60×60×60 cm) and was indirectly illuminated by two fluorescent lights. A 10 cm area near the surrounding wall was delimitated and considered the periphery. The rest of the open field was considered the central area. The distance travelled, the ratio of the distance travelled in the central area/total distance travelled, and the time in the center of the open- field were analyzed as a measure of anxiety-like behavior. During the test, mice were allowed to move freely around the open field and to explore the environment for 15 min.

### Isolation of cerebral microvessels

Cerebral microvessels were isolated from the remaining brain tissue as previously described by Zhang et al [45] with minor modifications. Brain tissue, devoid of large vessels, was homogenized in ice cold PBS with Dounce homogenizer and centrifuged twice at 2000 g at 4°C. The supernatant, containing the parenchymal tissue, was discarded. The pellet was resuspended in PBS and centrifuged as described above. The resulting pellet was resuspended and layered over 15% Dextran (in PBS) (Sigma, St.Louis, MO) and centrifuged at 4500 g for 30 minutes at 4°C. The top layer was aspirated and discarded and the remaining pellet resuspended in 15% Dextran and centrifuged. The final pellet was resuspended in 1% bovine serum albumin (BSA), the suspension was then passed though a 40-µm nylon mesh (BD Falcon). Microvessels retained on the mesh were washed with BSA/PBS and collected by centrifugation at 900 g for 10 minutes at 4°C.

### Data analysis

Values are shown as mean ± S.E.M in the text and figures. Differences between the groups were analyzed using one-way analysis of variance, followed by Bonferroni test. Probability values less than 0.05 were considered significant.

## Supporting Information

Figure S1 **Testes of SAMP8 and SAMR1 mice and role of nNOS in neuronal senescence.** **A.** Testis weight of SAMR1 and SAMP8 with or without testosterone. **B.** Photographs of SAMR1 donor and SAMP8 recipient mice. White arrows indicate operation scar. **C.** Expression of nNOS in MHC treated with resveratrol or testosterone under the oxidative stress. **D.** Activity of nNOS in MHC treated with resveratrol or testosterone under the oxidative stress. (^*^p<0.05, N = 3, n.s: not significant).
(TIF)
