# Supplementary figures and images for: Testosterone Deficiency Accelerates Neuronal and Vascular Aging of SAMP8 Mice: Protective Role of eNOS and SIRT1
Source: PLoS One. 2012 Jan 4;7(1):e29598. doi: 10.1371/journal.pone.0029598 (PMC3251570; doi:10.1371/journal.pone.0029598)

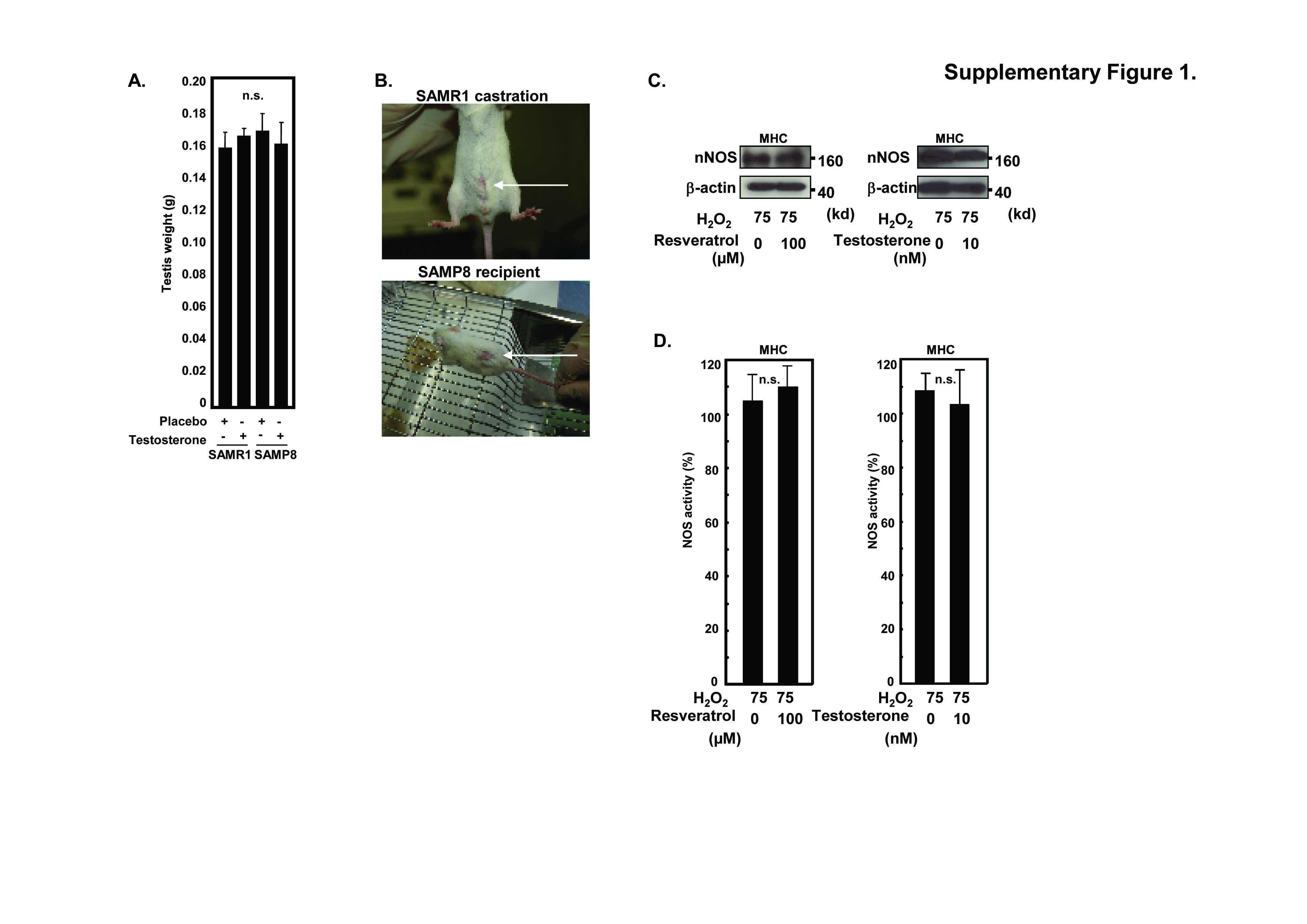

Supplement: Figure S1 — Testes of SAMP8 and SAMR1 mice and role of nNOS in neuronal senescence. A. Testis weight of SAMR1 and SAMP8 with or without testosterone. B. Photographs of SAMR1 donor and SAMP8 recipient mice. White arrows indicate operation scar. C. Expression of nNOS in MHC treated with resveratrol or testosterone under the oxidative stress. D. Activity of nNOS in MHC treated with resveratrol or testosterone under the oxidative stress. (*p<0.05, N = 3, n.s: not significant). (TIF) [file pone.0029598.s001.tif]
